# Shake and bake: a robust and cost-effective proteomic sample preparation workflow for plasma and cerebrospinal fluid

**DOI:** 10.1186/s12014-026-09589-1

**Published:** 2026-02-15

**Authors:** John Rönnholm, Sophia Weiner, Johannes Fuchs, Annika Thorsell, Carina Sihlbom, Laura Dugom, Amy Easton, Henrik Zetterberg, Johan Gobom

**Affiliations:** 1 Department of Psychiatry and Neurochemistry, Institute of Neuroscience & Physiology, the Sahlgrenska Academy at the University of Gothenburg, Mölndal, Sweden; 2 Clinical Neurochemistry Laboratory, Sahlgrenska University Hospital, Mölndal, Sweden; 3 Proteomics Core Facility, Sahlgrenska Academy, University of Gothenburg, Gothenburg, Sweden; 4 Target ALS Foundation, NY New York, USA; 5 Department of Pathology and Laboratory Medicine, University of Wisconsin School of Medicine and Public Health, Madison, WI USA; 6 Wisconsin Alzheimer’s Disease Research Center, School of Medicine and Public Health, University of Wisconsin, University of Wisconsin-Madison, Madison, WI USA; 7 Department of Neurodegenerative Disease, Institute of Neurology, University College London, London, UK; 8 Dementia Research Institute, University College London, London, UK; 9 Hong Kong Center for Neurodegenerative Diseases, Hong Kong, China; 10 Centre for Brain Research, Indian Institute of Science, Bangalore, India

**Keywords:** Plasma proteomics, CSF proteomics, Protein depletion, Sample preparation, DIA, TMT, Quantification, Neurodegenerative diseases

## Abstract

**Background:**

Plasma and cerebrospinal fluid are complementary sources of biomarkers for neurodegenerative diseases. The wide dynamic range of protein abundances, particularly in plasma, hampers detection of low-abundance proteins. Depletion of high-abundance proteins and efficient enzymatic digestion can improve proteome coverage but must be carefully optimized for reproducibility, throughput, and cost-efficiency for use in large-scale clinical proteomic studies.

**Methods:**

We developed a scalable sample preparation workflow for plasma and cerebrospinal fluid (CSF) that integrates depletion of high-abundance proteins, optimized digestion using Lys-C and trypsin, and compatibility with both label-free and tandem mass tag (TMTpro)-based quantification. Depletion was performed using a multi-affinity resin with immobilized antibodies targeting 14 high-abundance plasma proteins, which collectively constitute ≈ 95% of total plasma protein content. We systematically evaluated protein depletion and enzyme digestion conditions, and the effect of deoxycholate on digestion, monitoring the number of detectable proteins and the quantitation precision.

**Results:**

A resin-to-plasma ratio of ≥ 75 and a mixing speed of 900 rpm ensured complete and reproducible depletion. Depletion resulted in an increase in the number of identified proteins by ~ 65% in CSF, and ~ 80% in plasma, tripling the number of brain-enriched proteins, with maintained quantitative precision (median coefficient of variation (CV) for relative protein abundances < 11%). A two-step digestion protocol using Lys-C/trypsin followed by trypsin yielded the highest reproducibility and detectability in plasma. Adding the detergent deoxycholate to the samples had little effect in CSF and only marginally improved proteome coverage for plasma but decreased quantification precision and throughput. Technical replicates from a 528-sample clinical amyotrophic lateral sclerosis (ALS) cohort showed high reproducibility, with intra-sample CVs substantially lower than inter-individual variation.

**Conclusions:**

The sample preparation workflow described here enabled deep and reproducible proteome profiling of plasma and CSF in high-throughput formats and was found to be suitable for biomarker discovery in large clinical studies.

**Supplementary Information:**

The online version contains supplementary material available at 10.1186/s12014-026-09589-1.

## Background

Cerebrospinal fluid (CSF), which surrounds the brain and spinal cord, is in direct contact with the interstitial fluid of the central nervous system (CNS) and its protein composition can reflect a wide range of physiological and pathological CNS processes. Therefore, CSF is a valuable source of biomarkers of neurodegenerative diseases, increasingly applied in clinical diagnostics. However, the invasiveness of lumbar puncture limits the routine implementation of CSF biomarkers, particularly in primary care settings [1].

Blood plasma, in contrast, is readily accessible and contains many CNS-derived proteins, which may enter the circulation via multiple mechanisms, including blood-brain barrier disruption [2] or glymphatic and lymphatic clearance [3], active transport, or release due to neuroinflammation and cell damage. Blood-based biomarkers may facilitate the identification of patients developing neurodegenerative diseases, enabling timely medical intervention. One prominent example is plasma phospho-tau217, which has demonstrated high diagnostic accuracy for Alzheimer’s disease [4].

When searching for blood-based biomarkers of CNS diseases, it is informative to analyze both CSF and plasma proteomes. Being in closer proximity to the brain and spinal cord, CSF more directly reflects CNS pathology, making it a suitable reference for identifying candidate biomarkers. If the same proteins are detected in plasma and show correlated abundance profiles with those in CSF, this strengthens their potential as blood-based biomarkers as it suggests negligible peripheral contribution. In addition, plasma may contain biomarkers of peripheral origin that reflect downstream effects of CNS pathology, which could also hold diagnostic or prognostic value.

Several proteomic studies have used both label-free quantification and tandem mass tag (TMTpro)-based workflows to identify biomarkers in CSF and plasma [5–8]. However, the dynamic range of protein concentrations in these fluids, particularly the dominance of high-abundance blood proteins in plasma, poses a major analytical challenge, as these abundant proteins limit the sample volume equivalents that can be injected into LC–MS systems, thereby hampering the ability to detect lower-abundance proteins.

This problem can be addressed by depleting the most abundant protein, by using multi-affinity resins carrying immobilized antibodies targeting highly abundant blood proteins such as albumin, immunoglobulins, transferrin, haptoglobin, and complement factors [9–11]. Such resins are available in multiple formats, including LC columns, spin cartridges, and loose resin suitable for batch or plate-based workflows. Alternative strategies to decrease sample complexity are targeted enrichment methods [12–15] or partial protein precipitation [16–18]. These approaches serve to simplify the sample composition, enabling larger plasma volume equivalents to be used for analysis and thereby improving the ability to detect lower-abundant proteins. As such procedures affect the protein composition of the samples, it is important that they are performed reproducibly across all study samples in order not to introduce artifacts.

Achieving near complete and reproducible enzymatic digestion is also critical for ensuring accurate and consistent quantification. For large-scale biomarker discovery studies, both depletion and digestion protocols must be adapted for high-throughput, plate-based formats that can accommodate hundreds of clinical samples without compromising reproducibility.

In this study, we developed and systematically evaluated a depletion-based sample preparation workflow for plasma and CSF that is scalable and cost-efficient. We optimized critical steps in the workflow, including depletion efficiency, mixing conditions, digestion strategy, and the use of detergents, and evaluated their impact on protein identification and quantification using both experimental controls and clinical samples.

## Methods

### Aim and study design

**Fig. 1:**
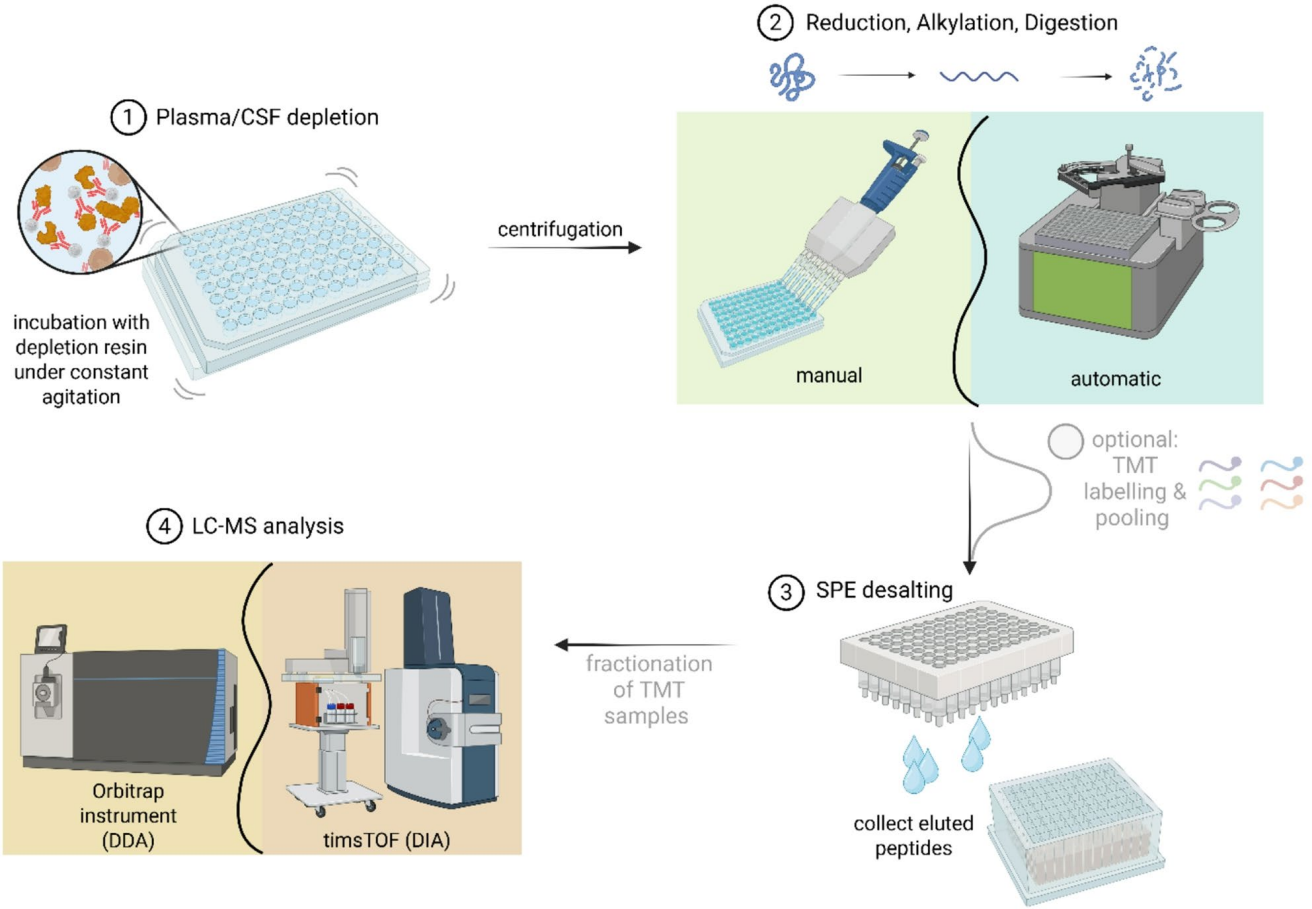
Sample preparation workflow [1]. High-abundance blood proteins were removed by incubation with multi-affinity resin in 96-well filter-bottom plates [2]. Reagents for reduction, alkylation, enzymatic digestion, and TMTpro labelling were added using an automated microdispenser [3]. Samples were desalted by SPE [4]. TMTpro multiplex samples were pre-fractionated by high-pH reversed-phase chromatography [5]. Samples were analyzed by Orbitrap (for TMTpro samples) or TIMS-TOF (for data-independent acquisition (DIA)) MS. Created with Biorender (Biorender.com)

Our aim was to develop a sample preparation regimen for both plasma and CSF that is scalable, cost-efficient, and compatible with both label-free quantification and quantification based on isobaric labelling using the tandem mass tag (TMT) technique. An overview of the workflow is shown in Fig. 1. Plasma and CSF samples were depleted of high-abundance proteins using multi-affinity removal resin, targeting 14 of the most abundant plasma proteins (Supplementary Material). Depletion was performed in 96-well filter-bottom plates, with flow-through collected by centrifugation. All sample preparation steps, including reduction of disulfide bonds, alkylation of cysteine residues, and enzymatic digestion using trypsin and Lys-C, were performed in solution. Reagent additions ≤ 10 µL were performed using a Mantis automated microdispenser (Formulatrix). For volumes > 10 µL, manual pipetting was used.

Following digestion, samples were either directly desalted by solid-phase extraction (SPE) for analysis by label-free quantification or labeled with proline-based tandem mass tags (TMTpro) for reporter ion-based quantification prior to desalting. To ensure high reproducibility and extensive proteomic coverage, we systematically evaluated key workflow parameters.

### Materials

Surplus patient CSF and plasma samples of unspecified clinical indication from the Clinical Neurochemistry Laboratory at Sahlgrenska University Hospital, Mölndal, were used for method development, after de-identification and pooling. CSF samples from ALS patients and healthy controls were obtained from the TargetALS foundation (https://www.targetals.org/). The ALS cohort was included exclusively for evaluation of technical reproducibility and scalability of the workflow, and not for biological or clinical interpretation of disease-associated protein changes.

### Total protein quantification

Total protein concentration in plasma was measured using kits from Bio-Rad (Protein Assay Kit) and Thermo Scientific (Micro BCA™ Protein Assay Kit), according to the manufacturers’ instructions.

### Depletion of plasma and CSF samples

Plasma samples (10 µL) were diluted in 330 µL of 10 mM phosphate-buffered saline (PBS). Aliquots of 20 µL diluted plasma were mixed with 50 µL of High-Select™Top14 Abundant Protein Depletion Resin (Thermo Scientific) in a 96-well polypropylene filter plate (AcroPrep Advance, 0.45 µM; Cytiva Life Sciences). CSF (50 µL, undiluted) was processed using 50 µL of depletion resin. Plates were incubated at room temperature (RT) on an orbital shaker (MTS 2/4, IKA) at 900 rpm for 45 min. Flow-through was collected by centrifugation at 1000 × g for 5 min into a 96-well polypropylene plate (Armadillo High-Performance PCR plate; Thermo Scientific). For some experiments, larger CSF input volumes (up to 75 µL) were used; in these cases, the volumes of reagents for reduction, alkylation, and proteolytic digestion were scaled accordingly.

### Reduction, alkylation, and proteolytic digestion

Depleted CSF samples were treated with 10 µL 4.5% sodium deoxycholate (DOC; Sigma); 450 mM tri-ethyl ammonium bicarbonate (TEAB; Sigma), followed by 5 µL 90 mM Tris [2]-carboxyethylphosphine (TCEP; Sigma) to reach final concentrations of 0.5% DOC; 50 mM TEAB; 5 mM TCEP. Depleted plasma samples were treated with 10 µL 3% DOC; 300 mM TEAB, followed by 5 µL 60 mM TCEP to reach final concentrations of 0.5% DOC; 50 mM TEAB; 5 mM TCEP. Samples were incubated for 1 h at 56 °C.

For alkylation, 5 µL iodoacetamide (IAA; Sigma) was added to a final concentration of 10 mM followed by incubating in the dark for 30 min at RT.

Proteolytic digestion was performed adding 5 µL of a mixture of endoproteinase Lys-C and trypsin (Promega), dissolved in resuspension buffer to a final concentration of 100 ng/µL for depleted plasma and 120 ng/µL for depleted CSF (estimated enzyme-to-protein ratio 1:10, w/w). Samples were incubated overnight at 37 °C. Depleted plasma samples were subjected to a second digestion using trypsin alone, added and incubated under the same conditions.

### Detergent removal and desalting for data-independent analysis

For DOC-containing depleted plasma samples, 1.5% trifluoroacetic acid (TFA; Fluka) was added to a final concentration of 0.1%, followed by 1 M hydrochloric acid (VWR) to pH ≤ 2 to precipitate the detergent. Samples were centrifuged at 2600 × g for 45 min at 4 °C to pellet the DOC.

Samples prepared without DOC were acidified with 1.5% TFA to pH ~ 3.

Desalting was performed using an Oasis HLB Prime 96-well plate (10 mg; Waters) and a vacuum manifold. Samples were loaded, washed twice with 500 µL of 5% methanol (Fisher Scientific) and peptides eluted with 1 mL 50% acetonitrile (ACN; Fisher Scientific) in 0.1% TFA. Eluates were collected in a polypropylene deep-well plate and lyophilized using a SpeedVac vacuum concentrator.

### TMTpro labeling and desalting

TMTpro reagents (5 mg; Thermo Scientific) were equilibrated to RT and dissolved in 200 µL ACN. A 7-µL (175 µg) aliquot was added to each sample, followed by incubation for 1 h at RT with shaking (900 rpm). Reactions were quenched with 5 µL of 4.48% hydroxylamine (Sigma) to a final concentration of 0.2%, followed by incubation for 30 min with constant agitation.

Labeled samples were combined into TMTpro multiplex samples and acidified to pH ≤ 2 by addition of 570 µL of 1 M HCl for CSF and 440 µL for plasma to precipitate DOC, followed by 4.5 mL 0.1% TFA for CSF and 5 mL for plasma to reduce the ACN concentration to < 3% prior to desalting. DOC was removed from the sample supernatants by centrifugation (2626 × g; 45 min; 4 °C). Desalting was performed using Sep-Pak C_18_ Light cartridges (Waters) and a vacuum manifold. This cartridge was used instead of the Oasis HLB 96-well plate (which was used for samples prepared for DIA) because of its higher protein binding capacity and loading volume capacity, to accommodate the TMT multiplex samples. Cartridges were activated twice with 1 mL of 80% ACN; 0.1% TFA and equilibrated twice with 1 mL of 0.1% TFA. Samples were loaded, washed twice with 1mL of 0.1% TFA, and peptides eluted with 1 mL of 80% ACN; 0.1% TFA.

### Offline high-pH reverse phase liquid chromatography sample fractionation

Peptide samples (TMTpro labeled samples and unlabeled samples used for spectral library generation) were fractionated at basic pH on an UltiMate™ 3000 LC system (Thermo Scientific). Samples were dissolved in 2.5 mM aqueous NH_4_OH and loaded onto an XBridge BEH C18 column (130 Å, 3.5 μm, 2.1 mm x 250 mm; Waters). Peptides labeled with TMTpro were separated with a linear increase of Buffer B (84% ACN) from 1% to 45% over 65-minutes, at a flow rate of 100 µL/min. Buffer C (25 mM NH_4_OH) was maintained constant at 10%. Fractions were collected every minute in a circular pattern across two rows in a deep 96-well plate (e.g., A1 → A12 → B12 → B1 → A1), yielding 24 concatenated fractions. For spectral library generation of depleted plasma, non-depleted plasma and non-depleted CSF, a 120-minute gradient at a flow rate of 150 µL/min was used. The gradient for buffer B was 1% (0 min), 20% (90 min) and 50% (120 min). Buffer C was maintained at 10%. Fractions were collected with a 25-second interval and concatenated into 48 fractionates by circling over four rows in a deep 96-well plate. The column was washed with 90% B/10% C for 10 min and equilibrated at 1% B/10% C for 10 min. Fractions were dried by vacuum centrifugation and stored at −20 °C pending LC–MS analysis.

### TimsTOF DIA LC–MS

timsTOF DIA analysis was performed on a timsTOF HT mass spectrometer (Bruker) coupled to an Evosep One (Evosep) liquid chromatography (LC) system. All samples were loaded onto Evotips Pure tips (Evosep) according to the manufacturer´s instructions. The LC system was operated using the 30 samples per day method (30SPD) on a Pepsep C18 column (15 cm x 150 μm ID, 1.5 μm; Bruker). Solvent A was 0.1% formic acid (FA) in MilliQ water, solvent B was 0.1% FA in ACN. The timsTOF HT was run in dia-PASEF mode using variable isolation windows. The isolation windows were created using py_diAID (version 0.018) [19] with the recommended default settings. The spectral library which was used for creating py diAID method was generated using timsTOF HT DDA runs of 48 high-pH fractions of crude and depleted plasma, CSF (data not shown). The cycle time was 2.73 s. The collision energy was set from 20 to 59 eV along an ion-mobility range of 0.6 to 1.6 Vs/cm^2^.

### Orbitrap LC–MS

Liquid chromatography–mass spectrometry (LC–MS) was performed using either a Tribrid Lumos Orbitrap MS coupled to an UltiMate RSLCnano HPLC system, or a Tribrid Eclipse Orbitrap MS coupled to a Vanquish Neo HPLC system (all from Thermo Scientific). The systems were used in different experiments as indicated in the text. Both systems operated in trap-and-elute mode, using PepMap Acclaim trap columns (300 μm × 5 mm) and EasySpray PepMap C18 analytical columns (75 μm × 500 mm; Thermo Scientific). Each MS system was equipped with a high-field asymmetric waveform ion mobility spectrometry (FAIMS) interface for gas-phase ion separation. On both systems, peptides were separated using a linear gradient of Buffer B (84% ACN, 0.1% formic acid): 4%−10% B over 1 min, followed by 10–40% over 65 min. The column was washed with 100% B for 3 min and re-equilibrated at 4% B for 13 min. Data-dependent acquisition (DDA) was performed in positive ion mode. MS1 scans were acquired in the Orbitrap (resolution: 120 k; scan range: m/z 400–1200; automatic gain control (AGC) target: 100%, maximum injection time: 50 ms) using a two-compensation voltage FAIMS method (−45 V and − 65 V), with a total cycle time of 3 s. Peptide fragmentation was performed by higher-energy collisional dissociation (HCD), and MS2 spectra were acquired the Orbitrap (resolution: 50 k for TMTpro 18-plex, 75 k for TMTpro 35-plex; AGC target: 200%; maximum injection time: 120 ms; isolation window = 0.7 m/z; activation type = HCD; collision energy = 32%).

### TMT data analysis

Protein identification and quantification was performed using Proteome Discoverer 3.2 (Thermo Scientific). The human subset of UniProtKB Swiss-Prot was searched using the SequestHT search engine with the following parameters: precursor Δm tolerance = 5 ppm, fragment Δm tolerance = 0.02 Da, missed cleavages = 2, minimum peptide length = 6, fixed modifications = carbamidomethylation, TMTpro (peptide N-terminus, Lys residues). Percolator was used for peptide scoring, filtering peptide spectral matches and peptides to a false discovery rate (FDR) of < 1%. The Most Confident Centroid integration method was used for peak integration for reporter ion quantification with integration tolerance set to 20 ppm. For TMTpro 35-plex quantification, A global internal standard (GIS; pool of CSF or plasma) was labeled with one deuterated and one non-deuterated TMTpro in each 35-plex set. Protein quantification was based on unique peptides. In the event of ambiguity, i.e., a peptide matching multiple proteins, peptides were assigned to a protein sequence in accordance with the principle of parsimony (shared peptide sequences are assigned to the most-supported protein). For data normalization, individual protein abundances were divided by that of their corresponding GIS (deuterated or non-deuterated). Each protein ratio was then additionally divided by the respective sample median, accounting for aberrant differences in total protein amount.

### DIA data analysis

The DIA data files were searched library-based in Spectronaut (19.4). For protein inference the reviewed human database (January 2024, 20428 entries) was used choosing only protein group specific as proteotypicity filter. Default settings were used for data analysis, but “run-level protein scoring” was set to “highest scoring observation”. Cross-run normalization was enabled.

### Generation of the spectral library

timsTOF HT DDA runs of fractionated CSF, depleted and undepleted samples were used to create the spectral library. The spectral library was created with fragpipe (19.1), MSFragger (3.8) and Philosopher (5.0.0) using default settings of the DIA_SpecLib_Quant workflow. Missed cleavages allowed were set to 1.

### Statistics

All statistical analyses were performed using R Statistics (v. 4.4.2). Differences in the mean number of protein groups were assessed using two-sample t-test for pairwise comparisons and ANOVA for comparisons across more than two groups.

## Results

### Depletion of high-abundance proteins

#### Determining the optimal resin-to-plasma-ratio

Given the high cost of multi-affinity depletion resin and to improve scalability in high-throughput workflows, we aimed to identify the lowest resin volume that maintained depletion efficiency. We incubated 20 µL of diluted plasma (corresponding to 0.59 µL neat plasma equivalent) with varying volumes of High-Select™ Top14 Abundant Protein Depletion Resin (Thermo Fisher Scientific) in a 96-well filter-bottom plate. Incubation was performed for 45 min at room temperature with agitation, and flow-through fractions were collected by centrifugation. Each resin-to-plasma ratio was tested in triplicate.

**Fig. 2:**
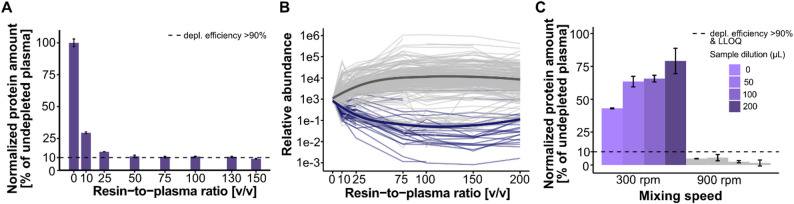
Effect of resin-to-plasma volume and mixing speed on immunodepletion in 96-well plates. (**A**) Plasma protein concentration (BCA assay; error bars indicate standard deviation) and (**B**) relative abundance (based on MS1 signal intensity) of identified plasma proteins after immunodepletion using different resin-to-plasma ratios. The dashed line indicates depletion efficiency of > 90%. (**C**) Plasma protein concentrations (BCA assay) obtained after incubation at different mixing speeds and sample dilutions with PBS. The dashed line indicates depletion efficiency of > 90% as well as the assay’s lower limit of quantification (LLOQ)

Bicinchoninic Acid (BCA) protein quantification revealed that at resin-to-plasma ratios of ≥ 50:1, the total protein content in the flow-through decreased to ~ 10% of that in neat plasma (Fig. 2A), with no further decrease observed at higher ratios. The coefficient of variation (CV) at a 50:1 ratio was 7.8%, and < 5% for higher ratios.

To evaluate effects on individual proteins, aliquots from each condition were analyzed by Orbitrap LC–MS using data-dependent acquisition (DDA), and protein abundances were quantified using MS1 precursor intensities (Fig. 2B). Of 277 quantified proteins, 22% (61 proteins) decreased in abundance with increasing resin volume, while 78% (216 proteins) increased. Among the 61 decreasing proteins, 44 matched the 14 proteins targeted for depletion and their known subclasses (Supplementary Table 1). Consistent with the BCA-based protein quantification data, protein-level depletion efficiency plateaued at resin-to-plasma ratios above 75:1.

### Effect of mixing speed and resin dilution on depletion efficiency

The manufacturer’s protocol for immunodepletion was designed for tube-based format using gentle end-over-end mixing. However, as this method is incompatible with 96-well plates, we instead performed incubation on a plate shaker. Suboptimal depletion observed in early experiments prompted us to evaluate the impact of mixing speed and resin dilution on depletion efficiency.

Plasma samples were incubated with a fixed amount of resin at a resin-to-plasma-ratio of 75:1 and shaken at either 300 or 900 rpm. To assess the effect of resin dilution, the resin was mixed with 0, 50, 100 or 200 µL PBS prior to incubation, yielding different post-centrifugation recovery volumes. Duplicate samples were prepared for each condition, and protein concentrations in the flow-through were measured by BCA assay (Fig. 2C).

At 300 rpm, depletion efficiency was limited, with only 20–60% of total protein removed, depending on the resin dilution. In contrast, shaking at 900 rpm resulted in > 90% depletion across all conditions. The effect of resin dilution decreased the depletion efficiency when shaking was performed at 300 rpm. The effect of resin dilution could not be determined at 900 rpm as the protein concentration fell below the assay’s lower limit of quantification (LLOQ). Since > 90% depletion was achieved at all dilutions, this parameter was deemed irrelevant at a mixing speed of 900 rpm.

An additional experiment in which bead incubation was performed with shaking at 800 rpm (Supplementary Fig. 1) resulted in similarly low depletion efficiency as observed at 300 rpm, indicating that a mixing speed of at least 900 rpm is required for efficient and reproducible depletion in a 96-well plate format.

### Effect of depletion on plasma proteome analysis

To evaluate the effect of depletion on plasma proteomic coverage, technical replicate samples were generated from a single depleted and a single non-depleted plasma preparation, respectively. Each preparation was digested once and subsequently aliquoted prior to DIA on a TimsTOF instrument. The optimal peptide input was estimated at 1 µg for depleted plasma and 0.5 µg for non-depleted plasma based on triplicate analyses (Supplementary Fig. 2).

**Fig. 3:**
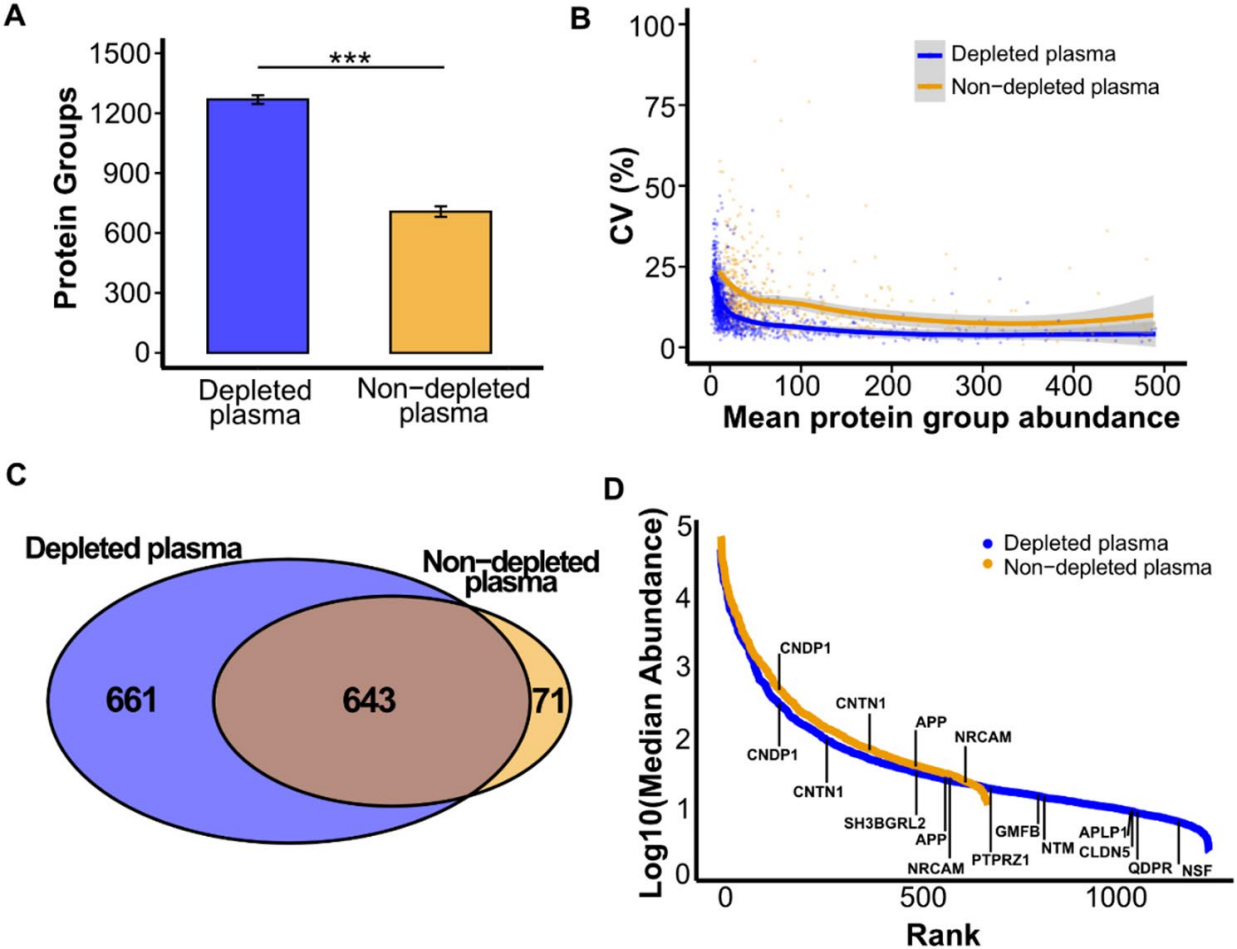
Effect of high-abundance protein depletion on plasma proteome coverage and quantification. (**A**) Average number of protein groups identified in depleted (blue) and non-depleted (orange) plasma. Statistical significance was assessed using a two-sample *t*-test. (**B**) Coefficient of variation (CV) for protein abundances plotted against mean abundance in depleted and non-depleted plasma. (**C**) Venn diagram showing the overlap of protein groups identified in depleted and non-depleted plasma. (**D**) Median protein abundances plotted in rank order. Protein groups predominantly expressed in the brain are highlighted. The relative abundance of each protein group was calculated as the mean across triplicate injections. A protein group was considered identified in a replicate only if detected in all three injections, and included in comparative analyses only if detected in at least half of the replicates

Depletion increased the average number of identified protein groups by approximately 80%, from 707 to 1269 (Fig. 3A; *P* < 0.0001). A protein group was defined as a set of proteins sharing the same set or subset of identified peptides. The CV for the number of protein group identifications was 1.7% for depleted plasma and 3.7% for non-depleted plasma. Quantification precision was comparable between conditions, with a median CV of 10.9% for depleted plasma and 11.0% for non-depleted plasma (Fig. 3B). Of the 714 protein groups identified in non-depleted plasma, 90% (643) were also detected in depleted plasma (Fig. 3C). Among the 71 protein groups that identified only in non-depleted plasma, 52 were immunoglobulins or immunoglobulin receptors targeted by the depletion resin, and two were hemoglobin subunits. The remaining 17 protein groups (FITM1, LRRTM2, EEF1E1, USP1, ANXA1, B4GALT1, LCN1, S100A7, KARS1, PTPA, TIGD7, BPIFB1, MUC16, ZG16B, PHOX2B, AKAP9 and TBC1D30) likely represent off-target protein losses. Conversely, protein groups uniquely identified in depleted plasma (Supplementary Table 2) were mostly lower-abundance proteins, including several with brain-enriched expression profiles (Fig. 3D).

### Effect of depletion on CSF proteome analysis

Depletion performance in CSF was evaluated using a TMTpro-based workflow, reflecting a common use case for CSF biomarker discovery studies. The plasma and CSF experiments were therefore not designed to enable a direct comparison between label-free DIA and TMT-based quantification, but rather to assess depletion performance under two complementary and widely used acquisition strategies.

To assess the effect of depletion on protein identification and quantification of CSF,18 aliquots of CSF (75 µL each) were processed with High-Select™ Top14 resin at a resin-to-sample ratio of 1:1, consistent with ratios used in previous studies [20, 21]. An additional 18 aliquots (25 µL each) were processed separately without depletion. Following digestion with trypsin and Lys-C, samples were labeled with TMTpro 18-plex reagents and combined into two multiplexed TMTpro sets. Each set was fractionated into 24 basic-pH reversed-phase fractions and analyzed by LC–MS on an Orbitrap mass spectrometer.

**Fig. 4:**
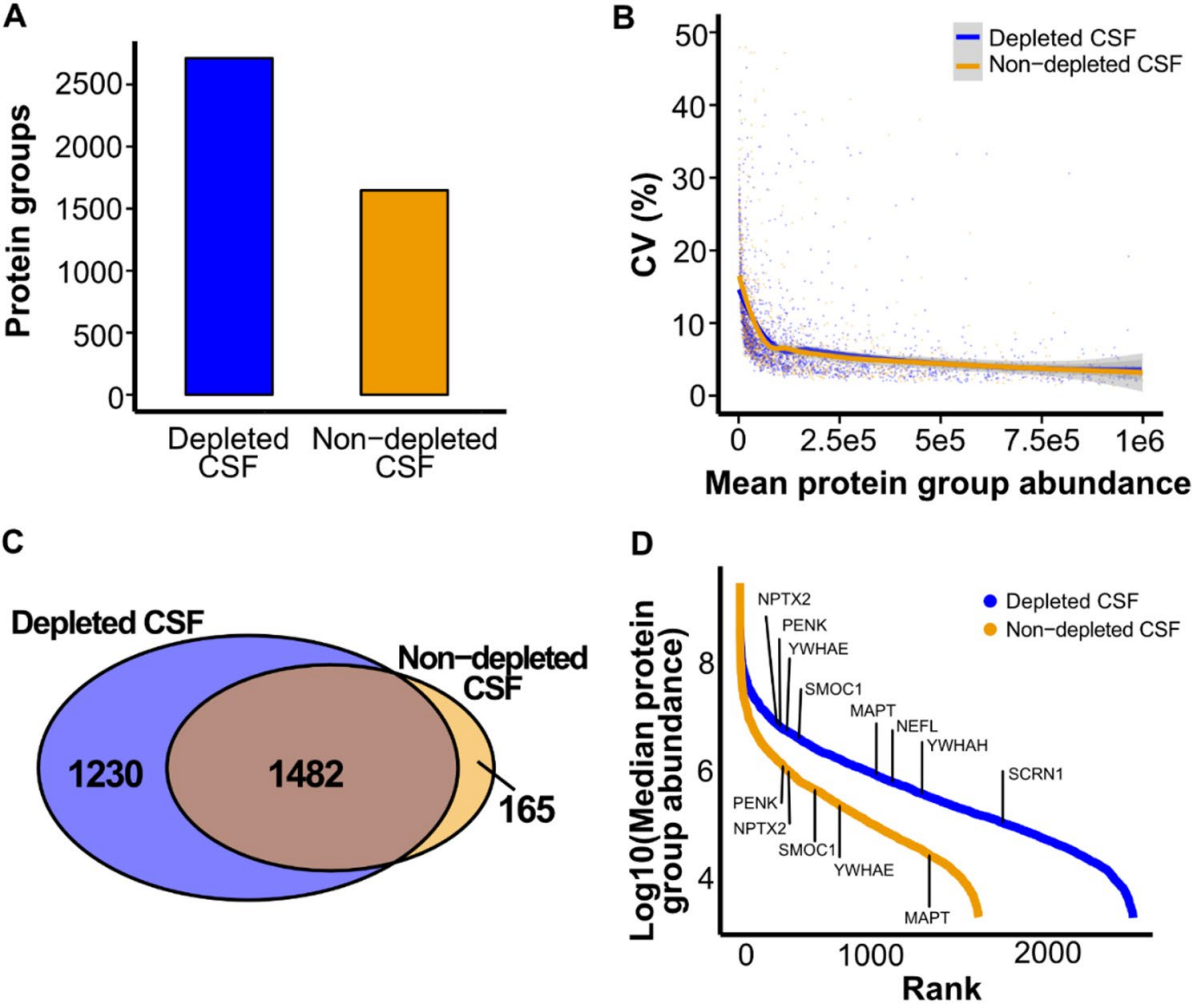
Effect of high-abundance protein depletion on CSF proteome coverage and quantification. (**A**) Number of protein groups identified in all 18 TMTpro channels without missing values, in depleted (blue) and non-depleted (orange) CSF. (**B**) Coefficient of variation (CV) in protein group abundance plotted against mean abundance for both conditions. (**C**) Venn diagram showing the overlap of protein groups identified in depleted and non-depleted CSF. (**D**) Median abundances of identified protein groups ranked by abundance. Proteins of interest in the context of neurodegenerative disease are highlighted [22–27]

Depletion increased the number of identified protein groups by 65%, from 1647 to 2712 (Fig. 4A). Protein groups were considered identified only if detected in all TMTpro channels (i.e., no missing values). Quantitative reproducibility was similar between conditions, with mean CVs of 4.7% for depleted CSF and 5.9% for non-depleted CSF (Fig. 4B).

Of the protein groups identified in non-depleted CSF, 90% (1482 of 1647) were also identified in depleted CSF (Fig. 4C). Among the 165 protein groups identified exclusively in non-depleted CSF, 49 were immunoglobulins targeted for removal, and 10 were keratins likely introduced as contaminants. The remaining proteins are listed in Supplementary Tables 3, and the protein groups only identified in depleted CSF in Supplementary Table 4. Depletion reduced the dominance of high-abundance proteins and resulted in a more even distribution of signal intensities across the dynamic range, facilitating detection of low-abundance proteins (Fig. 4D).

### Protein digestion

#### Protease selection and addition

To assess whether combined digestion with trypsin and Lys-C improves proteome coverage and quantification reproducibility in plasma, we compared three protocols for protein digestion in depleted plasma: (i) single-step digestion with a Lys-C/trypsin mixture; (ii) two-step digestion with Lys-C/trypsin followed by a second incubation with trypsin; and (iii) single-step digestion with trypsin alone. All enzymes were added at a 1:10 enzyme-to-protein ratio, and incubations were performed overnight at 37 °C.

**Fig. 5:**
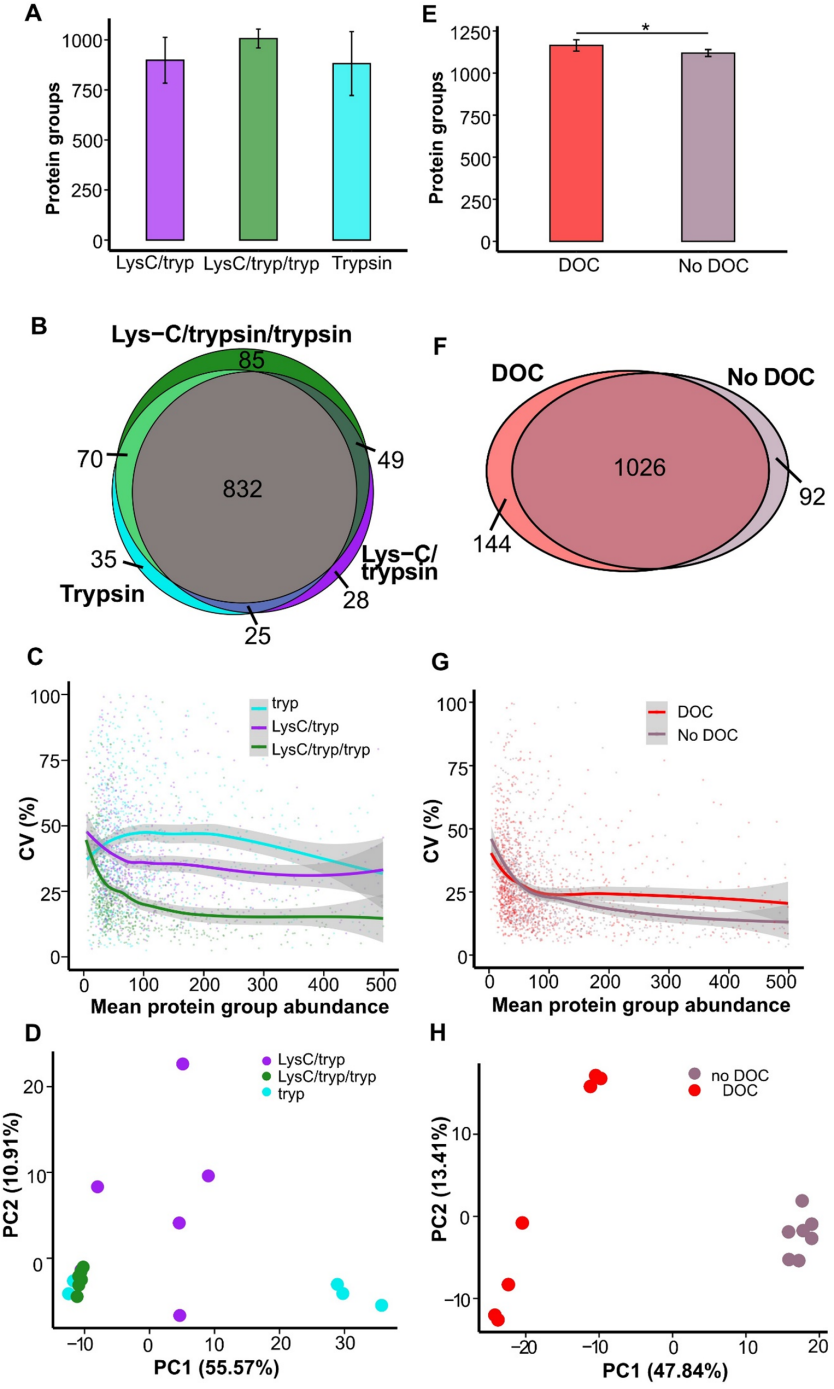
Effect of protease digestion strategy and sodium deoxycholate (DOC) on protein identification and quantification in plasma. (**A**–**D**) Comparison of three digestion protocols applied to depleted plasma: (i) single-step digestion with trypsin and Lys-C, (ii) two-step digestion with trypsin/Lys-C followed by a second incubation with trypsin, and (iii) single-step digestion with trypsin. (**A**) Number of identified protein groups across replicates. (**B**) Venn diagram showing the overlap of protein groups across the three protocols. (**C**) Distribution of protein-level CVs across replicates. (**D**) PCA scores plot based on protein abundances, showing replicate clustering for each digestion protocol. (**E**–**H**) Comparison of samples digested with protocol (iii) in the presence or absence of 0.5% DOC. (**E**) Number of identified protein groups. (**F**) Venn diagram showing protein group overlap between DOC-treated and untreated samples. (**G**) Distribution of protein-level CVs across replicates. (**H**) PCA scores plot comparing replicate clustering between DOC-treated and untreated samples

Six replicate digests per protocol were analyzed using timsTOF MS. The total number of identified protein groups (detected in at least half of the replicate runs) did not differ significantly between protocols (*P* > 0.05). However, protocol (ii), involving a two-step digestion, showed markedly lower variability in protein group identification across replicates (CV 4.7%) compared to protocol (i) (CV 12.7%) and protocol (iii) (18.1%; Fig. 5A).

Across all three protocols, 85% (832 of 980) of identified protein groups were shared, while 15.1% (148) were detected exclusively in a single condition (Fig. 5B). Protocol (ii) also yielded the highest quantitative reproducibility, with a median CV of 19.8%, compared to 32.2% for protocol (i) and 42.0% for protocol (iii) (Fig. 5C). Principal component analysis (PCA) further supported improved consistency for protocol (ii), with tighter clustering along PC1 and PC2 in the scores plot (Fig. 5D).

#### Effect of deoxycholate

To evaluate the effect of sodium deoxycholate (DOC) on protein identification and quantification, depleted plasma aliquots were digested using protocol (ii) (Lys-C/trypsin followed by trypsin) in the presence or absence of 0.5% DOC (w/v) in the digestion buffer. Each condition was prepared in seven replicates and analyzed by timsTOF MS.

A marginal but statistically significant difference was observed in the number of identified protein groups (*P* < 0.05), with an average of 1165 proteins (CV: 2.7%) identified in DOC-treated samples compared to 1119 proteins (CV: 1.8%) without DOC (Fig. 5E). Protein groups were considered identified if detected in at least four out of seven replicates.

The two conditions showed 81.3% overlap, with 1026 protein groups identified in both (Fig. 5F). Among these shared proteins, 67 exhibited significantly increased signal intensity in DOC-treated samples (*P* < 0.05), including 10 serine protease inhibitors. In contrast, 465 shared protein groups showed decreased signal intensity in DOC-treated samples (Supplementary Fig. 3).

Quantification precision was higher in samples without DOC, with a median CV of 20.4% compared to 24.7% in DOC-treated samples (Fig. 5G). Principal component analysis (PCA) further supported improved reproducibility without DOC, as untreated samples showed tighter clustering in the PC1–PC2 space (Fig. 5H).

#### Overlap of the CSF and plasma proteomes

**Fig. 6:**
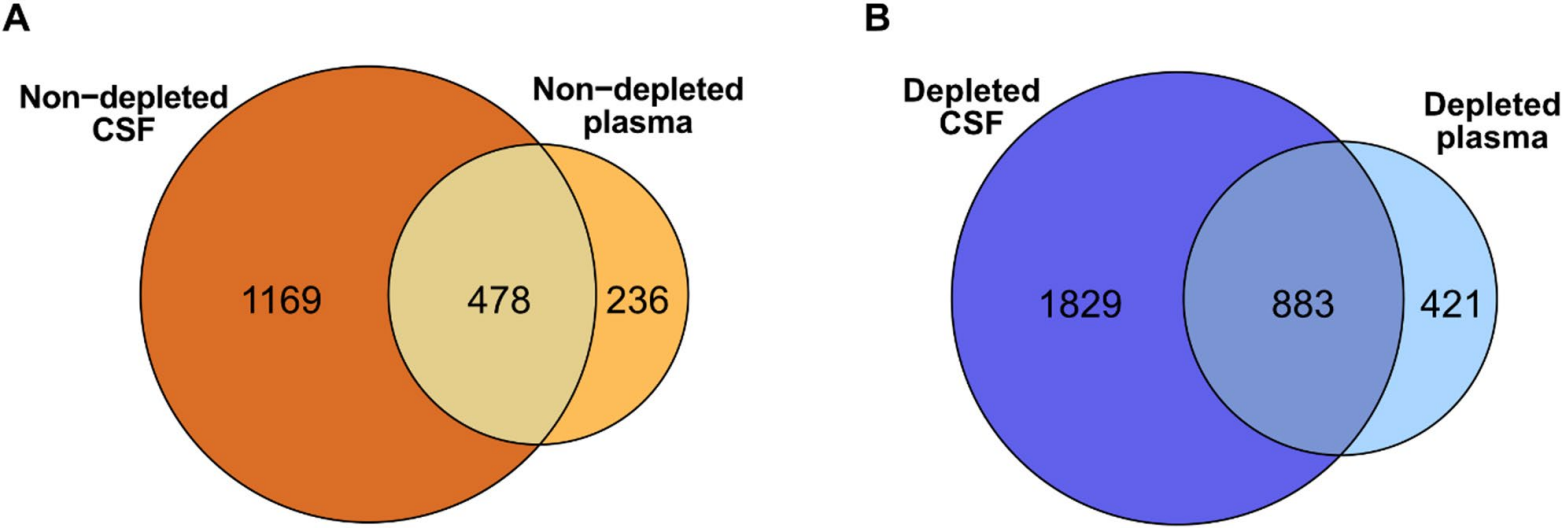
Overlap of protein groups identified in CSF and plasma. (**A**) Venn diagram showing the overlap of protein groups identified in non-depleted CSF and plasma. (**B**) Overlap of protein groups identified in depleted CSF and plasma

Of the 714 protein groups identified in non-depleted plasma, 478 (66.9%) were also detected in non-depleted CSF (Fig. 6A). In depleted plasma, 883 protein groups (67.7% of the 1304 detected proteins) were also detected in depleted CSF (Fig. 6B). To assess whether depletion increases the likelihood of detecting CNS-derived proteins in plasma, we performed tissue expression analysis of the overlapping proteins identified with and without depletion, using data from the Human Protein Atlas [28] (Supplementary Material).

Among the proteins shared between non-depleted plasma and CSF, 262 were classified as highly expressed in all tissues except brain (Category i), while 83 proteins showed high expression in the brain but were also expressed in peripheral tissues (Category ii). Only four protein groups (*APP*, *CNDP1*, *CNTN1*, and *NRCAM*) were classified as highly expressed exclusively in brain tissue (Category iii).

Following depletion, the number of overlapping proteins increased across all expression categories, with 556 proteins in category i (highly expressed in all tissues except brain), 187 in category ii (highly expressed in brain but also present in peripheral tissues), and 12 in category iii (exclusively expressed in brain). The eight additional brain-enriched proteins uniquely detected in depleted plasma were APLP1, CLDN5, GMFB, NSF, NTM, PTPRZ1, QDPR, and SH3GRL2. The median abundance of category iii proteins is highlighted in Fig. 3D, while the tissue distributions of all overlapping CSF and plasma proteins is shown in Supplementary Figs. 4 A (depleted CSF/plasma) and 4B (non-depleted CSF/plasma).

#### Evaluation of technical reproducibility in a clinical study

The technical reproducibility of the method was evaluated in clinical proteomic study of amyotrophic lateral sclerosis (ALS) using TMTpro 35-plex, comprising 231 CSF and 297 plasma samples from ALS patients and healthy controls obtained through the Target ALS Longitudinal Biofluid Core(full data set available via Target ALS (https://dataengine.targetals.org/collections). The CSF and plasma samples originated from 90 to 100 individuals, respectively, with several participants sampled on multiple occasions. Among the samples were 19 CSF and 25 plasma technical replicate pairs.

**Fig. 7:**
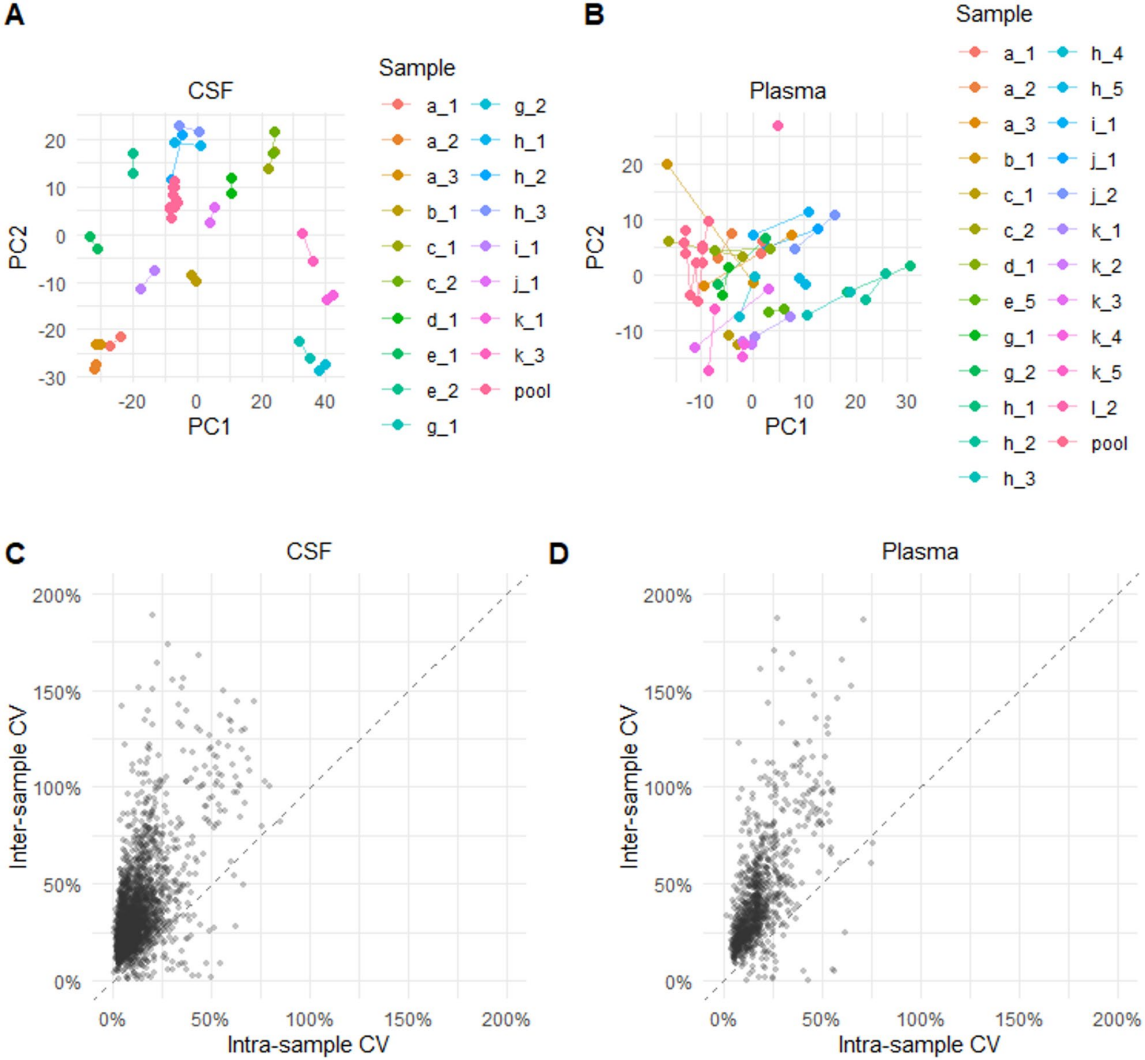
Evaluation of technical reproducibility in CSF and plasma proteomics. (**A**–**B**) Principal component analysis (PCA) of CSF (**A**) and plasma (**B**) protein abundance profiles, based on 2,939 quantified proteins. In the score plots, each individual is labeled by a letter (a–k), and sampling time points are indicated by numbers [1–5]. Technical replicate pairs are connected by lines. Technical replicates cluster closely in PC1–PC2 space, whereas longitudinal samples from the same individual and samples from different individuals show greater dispersion. (**C**–**D**) Scatterplots of intra-sample versus inter-sample coefficients of variation (CVs) for all quantified proteins in CSF (**C**) and plasma (**D**). Intra-sample CVs were calculated across 19 CSF and 25 plasma technical replicate pairs. Mean intra-sample CVs were 13% (CSF) and 19% (plasma), compared with mean inter-sample CVs of 34% and 42%, respectively.

Principal component analysis (PCA) of the CSF proteomic data (Fig. 7A) showed that technical replicates clustered tightly in PC1-PC2 space, compared with replicate pairs from different individuals and to longitudinal samples from the same individual. The same trend was observed for plasma (Fig. 7B), although the clustering of technical replicates was less distinct; an observation that may partially be explained by the higher missingness in plasma protein data set compared with CSF (Supplementary Fig. 5).

CVs were calculated across all proteins (2939 for CSF and 1139 for plasma), comparing both inter-sample and intra-sample variation in CSF (Fig. 7C) and plasma (Fig. 7D). In CSF, the mean inter-sample CV was 34% and the mean intra-sample CV 13%. For plasma, the corresponding CVs were 42% and 19%, respectively.

## Discussion

In this study, we developed and systematically evaluated a scalable, cost-effective, and reproducible workflow for proteomic profiling of plasma and CSF. The protocol incorporates high-abundance protein depletion, optimized enzymatic digestion, and compatibility with both label-free and TMTpro-based quantification strategies.

Several alternative strategies to protein depletion have been proposed to increase proteome coverage in plasma and CSF, including nanoparticle-based protein corona enrichment and commercial bead-based kits. Studies using these strategies have reported coverage ranging from ~ 1,500 to more than 4,000 plasma proteins [13, 14, 29]. These techniques typically rely on proprietary reagents and dedicated automation platforms, with costs of 80–90 USD per sample. Moreover, because nanoparticle corona formation is governed by affinity rather than selective removal of intact proteins, the resulting enrichment may contain a mixture of full-length proteins and proteolytic fragments, which can complicate biological interpretation. Acid precipitation methods, using perchloric acid or trichloroacetic acid, provide a cheaper alternative and have also been explored, either alone or in combination with immunodepletion [16–18]; however, this approach may also have undesired selectivity, potentially favoring enrichment of smaller, soluble protein degradation products, and reproducibility can be sensitive to experimental conditions. In contrast, immunodepletion using a multi-affinity resin provides a simple and widely accessible means of reducing dynamic range, with a per-sample cost of 3–4 USD in our hands, and with the advantage that depletion removes a defined set of well-characterized high-abundance proteins.

Initial sample volume is an important practical consideration in clinical proteomics, particularly for longitudinal studies and biobanked material. The present workflow requires 10 µL of plasma and 50–75 µL of CSF prior to dilution and depletion, which is well within the range typically available in clinical cohorts. Commercial enrichment-based workflows, such as bead-based plasma enrichment kits, are often optimized for similarly low plasma input volumes, commonly on the order of ~ 10–20 µL per sample, although reported performance depends on enrichment chemistry and downstream analytical depth. Thus, the starting material requirements of the present depletion-based workflow are comparable to those of targeted enrichment approaches.

While enrichment- and depletion-based strategies can be considered complementary, our goal in this study was to establish a cost-efficient, scalable, and open workflow suitable for large clinical cohorts across both plasma and CSF. The present optimization of resin usage, mixing conditions, and digestion strategy fulfills this need while delivering substantial gains in proteome coverage and quantitative reproducibility.

Reducing the background of high-abundance blood-derived proteins improves detection of CNS proteins, particularly in plasma. High-Select™ Top14 is one of several commercially available multi-affinity depletion products, which has been used in several plasma proteomic studies [6, 8, 17, 20, 30]. Due to its high cost, we aimed to minimize resin usage without compromising performance. We found that a resin-to-plasma volume ratio of ≥ 75:1 was sufficient for efficient and consistent depletion, removing approximately 90% of total protein. Depletion efficiency plateaued at this ratio, with CVs below 5% and no additional protein removal at higher resin volumes. This ratio is somewhat higher than the 40:1 ratio reported by Zhou et al. [17]. However, depletion efficiency in that study appears to have been slightly lower (80–85%; 12 g/L from an initial 60–80 g/L), indicating that the higher ratio achieves more complete depletion.

Efficient mixing was found to be critical for reproducible depletion in 96-well plates. While the original manufacturer protocol recommends end-over-end tube rotation, this method is incompatible with plate-based workflows. Our results showed that mixing at 900 rpm on a plate shaker was necessary to achieve consistent depletion efficiency. At lower speeds (300–800 rpm), resin sedimentation led to poor and variable depletion, emphasizing that mixing speed is a critical parameter when adapting depletion to high-throughput formats.

In both plasma and CSF, depletion substantially increased proteome coverage. In plasma, the number of identified protein groups increased by ~ 80%, and in CSF by 65%. Notably, several brain-derived proteins were exclusively detected in depleted plasma and disease-relevant proteins were identified only in depleted CSF. For example, neurofilament-light polypeptide (NEFL), was only identified in CSF after depletion, while microtubule-associated protein tau (MAPT), increased 30-fold in abundance after depletion. While most proteins appeared unaffected by depletion, a small number of proteins not targeted by the resin were lost. These off-target effects, observed in both plasma and CSF, were relatively limited but may affect quantification. Such losses should be considered when applying depletion strategies in targeted assays or absolute quantification workflows.

Near-complete and reproducible proteolytic digestion is essential for accurate protein quantification in MS–based proteomics. Initial experiments performed using a trypsin-to-protein ratio of 1:20 (w/w) for plasma digestion indicated incomplete proteolysis, prompting us to evaluate increasing protease amount and the benefit of a second digestion step. The use of trypsin in combination with endoproteinase Lys-C has been shown to enhance protein digestion by reducing missed cleavages and increasing protein identifications [31]. In the present study, plasma samples benefited from a two-step digestion strategy, using first a Lys-C/trypsin mixture followed by a second incubation with trypsin. In contrast, CSF exhibited low and expected levels of missed-cleavages after a single Lys-C/trypsin digestion, indicating that a second digestion step was unnecessary. This difference is likely attributable to the substantially higher total protein concentration in plasma and the presence of abundant endogenous protease inhibitors, such as α1-antitrypsin and α2-macroglobulin, which are present at much lower concentrations in CSF. These factors can limit digestion efficiency in plasma and are mitigated by a second digestion step, while a single digestion was sufficient for CSF.

DOC is an ionic detergent commonly used to disrupt protein interactions and partially denature proteins, thereby enhancing enzymatic digestion efficiency [32]. After digestion, DOC can be precipitated by acidifying the samples and removed by centrifugation. However, recovering the supernatant requires careful manual pipetting, which is impractical for large-scale, label-free studies and may impair reproducibility.

In our experiments, DOC increased the mean number of identified protein groups in depleted plasma by 4%. Of the protein groups identified with or without DOC, a majority (81%) were detectable under both conditions. Only 67 protein groups had significantly higher abundance in DOC treated samples, including 10 serine protease inhibitors, consistent with the known resistance of these proteins to cleavage by trypsin under native conditions. In contrast, 465 protein groups were more abundant without DOC, which may reflect sample loss during supernatant recovery or ion suppression due to incomplete detergent removal. DOC-treated samples also showed decreased quantitative reproducibility, with a median CV of 25% compared to 20% without DOC, and exhibited more dispersion in PCA space. Thus, while DOC marginally increases proteome coverage, this benefit may be outweighed by lower reproducibility and reduced compatibility with high-throughput workflows.

Primary biomarkers of CNS diseases are likely found in the overlap between the CSF and plasma proteomes. In our study, approximately two-thirds of the proteins identified in plasma were also detected in CSF, regardless of depletion. However, the number of overlapping protein groups increased substantially with depletion, from 478 to 882. Importantly, the number of brain-enriched proteins (Human Protein Atlas category iii) identified in plasma increased from 4 to 12 following depletion, underscoring the improved detectability of low-abundance CNS-derived proteins. This finding supports the use of depletion strategies to enhance biomarker discovery in plasma for neurodegenerative diseases.

The observation that samples from the same individual clustered together, relative to samples from different individuals, along PC1 and PC2 in PCA of the proteomic data in the ALS study indicates that the technical variation introduced by sample preparation and LC–MS analysis was smaller than the biological variation. This supports the feasibility of using these data to detect disease-associated biomarkers in large-scale clinical studies. Importantly, the ALS cohort data presented here are intended to demonstrate the technical robustness and scalability of the workflow by contrasting technical and inter-individual variability, rather than to support biological or clinical interpretations of disease-associated protein changes. Apart from the described ALS study, this sample preparation was used in a recent CSF proteomic study of Huntington’s disease [27].

The clustering was more distinct in CSF than in plasma. One possible explanation is that plasma proteins have a wider concentration span than CSF, ranging over 10–12 orders of magnitude, potentially causing more ion suppression that could lead to larger run-to-run variation. Indeed, a higher missingness was observed in the plasma data than for CSF. It is also possible that data normalization works better in CSF, having fewer proteins with extremely high concentrations.

## Conclusions

We developed and optimized a high-throughput sample preparation workflow for proteomic profiling of plasma and CSF, integrating high-abundance protein depletion, reproducible enzymatic digestion, and compatibility with both label-free and TMTpro-based quantification. A resin-to-plasma ratio of ≥ 75 and a plate shaker speed of 900 rpm were identified as critical parameters for achieving efficient and consistent depletion in 96-well format. Depletion significantly increased proteome coverage in both plasma and CSF without compromising quantification precision, and enabled detection of additional brain-enriched proteins in plasma. A two-step digestion strategy using Lys-C and trypsin yielded the highest reproducibility in plasma, while sodium deoxycholate slightly improved proteome coverage, albeit at the expense of quantitative precision and workflow scalability. The protocol demonstrated good technical reproducibility in a clinical cohort of over 500 samples, with analytical variation consistently lower than biological variation. This cost-effective and scalable workflow supports large-scale biomarker discovery efforts and is well suited for clinical proteomics studies focused on neurodegenerative diseases.

## Supplementary Information

Supplementary Material 1

Supplementary Material 2

Supplementary Material 3

## Data Availability

MS data from the clinical study of ALS can be obtained through the Target ALS foundation (full data set available via Target ALS: [https://www.targetals.org/](https:/www.targetals.org)).DIA data, spectral library and Spectronaut analysis files have been deposited to the MassIVE repository with the dataset identifier MSV000099682.

## Abbreviations

CSF: Cerebrospinal fluid
CNS: Central nervous system
TMTpro: Tandem mass tag with isobutyl proline reporter group
SPE: Solid-phase extraction
PBS: Phosphate-buffered saline
RT: Room temperature
DOC: Deoxycholate
TEAB: Tri-ethyl ammonium bicarbonate
TCEP: Tris(2)-carboxyethylphosphine
IAA: Iodoacetamide
TFA: Trifluoroacetic acid
CAN: Acetonitrile
LC–MS: Liquid chromatography–mass spectrometry
DDA: Data-dependent acquisition
FAIMS: High-field asymmetric waveform ion mobility spectrometry
AGC: Automatic gain control
HCD: Higher-energy collisional dissociation
BCA: Bicinchoninic acid
LLOQ: Lower limit of quantification
PCA: Principal component analysis
