# Supplementary material for: Shake and bake: a robust and cost-effective proteomic sample preparation workflow for plasma and cerebrospinal fluid
Source: Clin Proteomics. 2026 Feb 15;23:12. doi: 10.1186/s12014-026-09589-1 (PMC12927225; doi:10.1186/s12014-026-09589-1)
Supplement: Supplementary file 2 — Supplementary Material 2 [file 12014_2026_9589_MOESM2_ESM.docx]

**Supplementary Figure 1.** Effect of mixing speed on protein depletion efficiency. Incubation of plasma with bead resin (sample-to-bead resin volume ratio 1:75) was performed for 30 min at 800 and 900 RPM, respectively (n=4). The protein concentration was determined using a BCA assay.


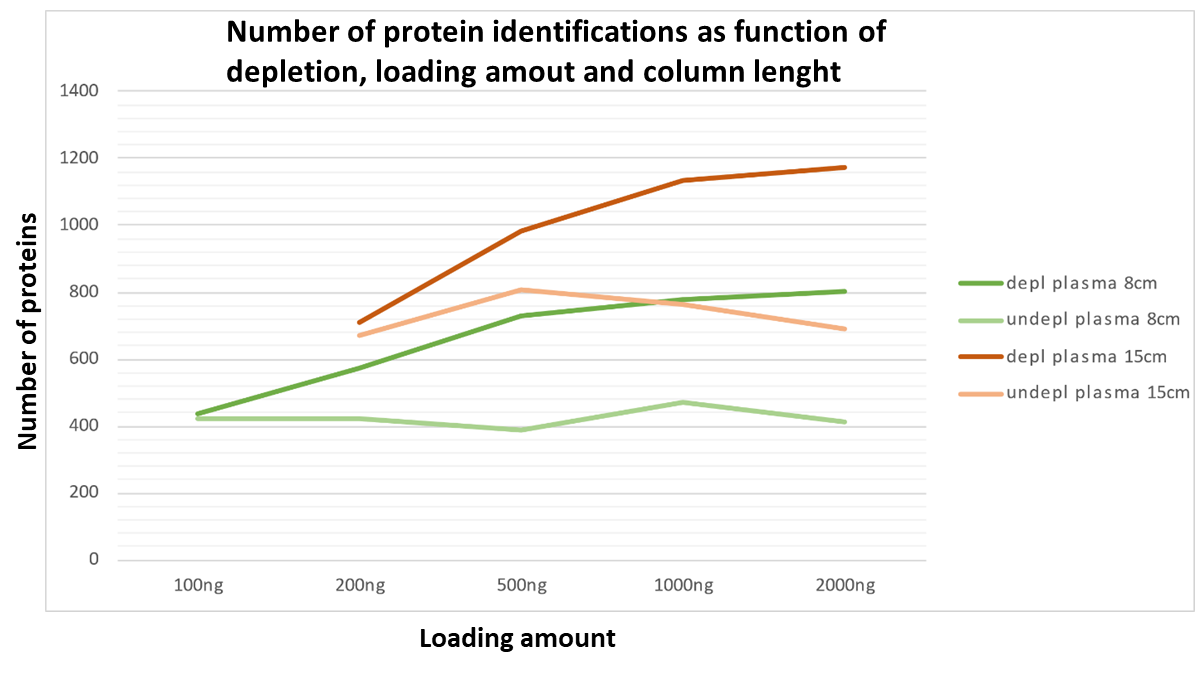


**Supplementary Figure 2.** Number of protein groups identified by data-independent acquisition (DIA) on a timsTOF instrument from depleted and non-depleted plasma across different peptide loads and chromatographic separation lengths (8-cm and 15-cm columns). Values represent the mean of three technical replicate injections.


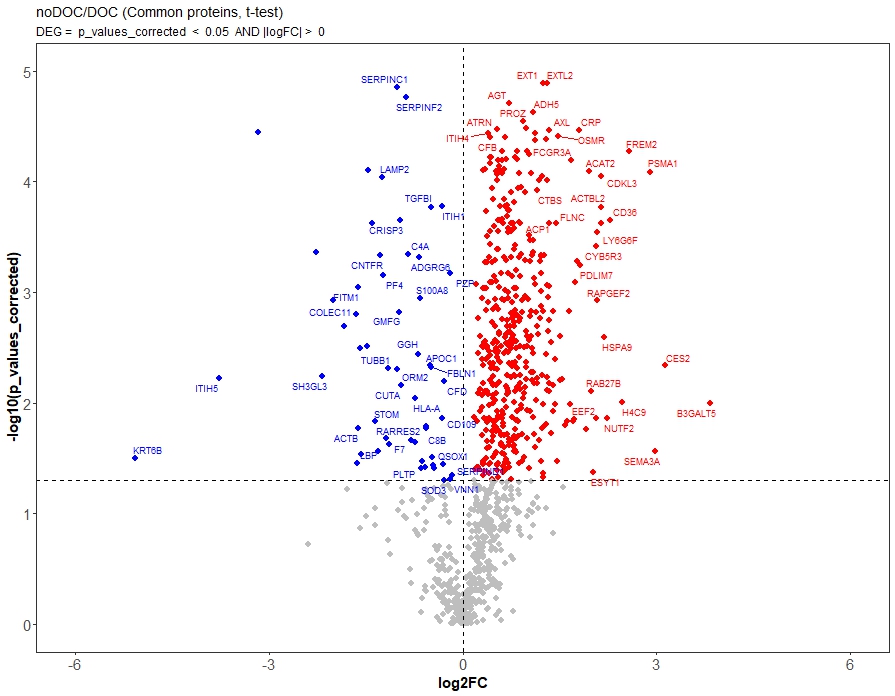


**Supplementary Figure 3.** Volcano plot of common protein groups identified with and without DOC. Only proteins with at least four observations were included in the analysis. Data were log₂-transformed and differences between groups were assessed using a two-sample *t*-test. The x-axis represents the log₂ fold change and the y-axis shows the –log₁₀ *p*-values.


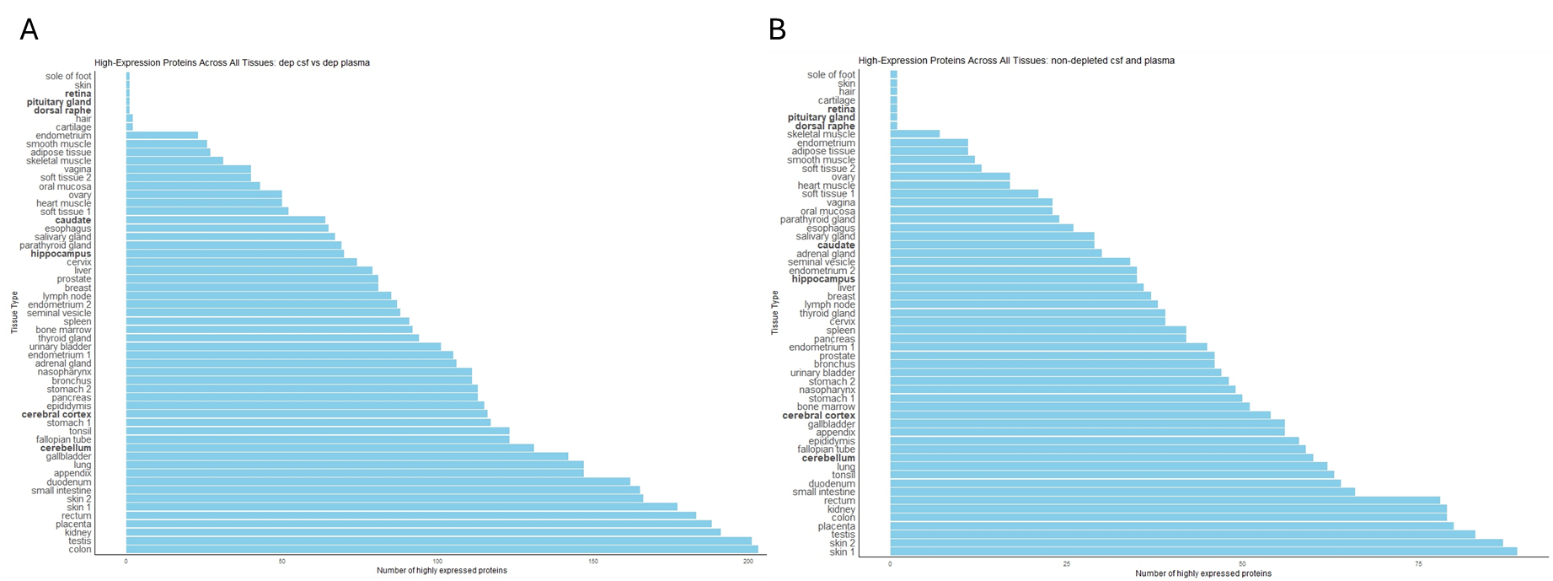


**Supplementary Figure 4.** Tissue expression distribution of proteins detected in both CSF and plasma following depletion (A) and without depletion (B). Brain tissues are indicated in bold.

**Supplementary Figure 5.** Completeness curves for CSF and plasma proteins, respectively, in the ALS cohort. Completeness is defined as the fraction of study participants in which a given protein was quantified.
