# Supplementary material for: Shake and bake: a robust and cost-effective proteomic sample preparation workflow for plasma and cerebrospinal fluid
Source: Clin Proteomics. 2026 Feb 15;23:12. doi: 10.1186/s12014-026-09589-1 (PMC12927225; doi:10.1186/s12014-026-09589-1)
Supplement: Supplementary file 3 — Supplementary Material 3 [file 12014_2026_9589_MOESM3_ESM.docx]

Shake and bake: a cost-effective sample preparation recipe for increased proteome coverage and reproduciblity in plasma and CSF – Supplementary material

# Methods

## Notes

### Depletion resin

Protein depletion was performed using High-Select™ Top14 Abundant Protein Depletion Resin
(Thermo Scientific, Product Number A36372). The resin carries immobilized antibodies targeting the following abundant blood proteins: Albumin, IgA, IgD, IgE, IgG, IgG (light chains), IgM, Alpha-1-acid glycoprotein, Alpha-1-antitrypsin, Alpha-2-macroglobulin, Apolipoprotein A1, Fibrinogen, Haptoglobin, Transferrin.

### Depletion

In the experiment described in Figure 3, depletion was performed by mixing 7.5 mL depletion resin with 100 µL plasma in a 10 mL column body. The mixture was then incubated on an end-over-end shaker for 30 minutes. In the experiment described in Figure 5, 10 µL plasma was diluted in 670 µL of 10 mM Phosphate-buffered saline (PBS). Then, 20 µL of diluted plasma, 50 µL depletion resin and optionally 25 µL of PBS were added to an AcroPrep Advance 96-well plate (0.45 μM, Cytiva). Flow-through was collected by centrifugation at 1000 × g for 5 minutes into a 96-well polypropylene plate an (Armadillo High-Performance PCR plate; Thermo Scientific).

### Reduction

In the experiments described in Figure 2, 3, 5, and 6, reduction of cysteine disulfide bonds in plasma samples were performed using dithiothreitol (DTT) according to the protocol below. In the clinical ALS study (Figure 7), Tris(2)-carboxyethylphosphine (TCEP) was used, same as for CSF. No difference in protein identification and quantification results was observed between the two reducing agents.

Reduction with DTT:

To reduce disulfide bonds in depleted plasma samples, 50 mM dithiothreitol (DTT; Roche), 500 mM triethylammonium bicarbonate (TEAB; Sigma) and, optionally, 5% sodium deoxycholate (DOC; Sigma) were added to reach final concentrations of 5 mM DTT, 50 mM TEAB and 0.5% DOC. Samples were incubated for 1 hour at RT on an orbital shaker.

### Tissue expression analysis of overlapping plasma and CSF proteins

Tissue expression profiles were obtained from the Human Protein Atlas (<https://www.proteinatlas.org/>). The protein groups were filtrated by “high expression” as well as by reliability ratings of “approved”, “supported” and/or “enhanced”. Protein groups with high expression in several cell types within the same tissue were simplified to a single tissue-level classification. The following tissues were categorized as brain: "caudate", "cerebellum", "cerebral cortex", "hippocampus", "hypothalamus", "choroid plexus", "dorsal raphe", "substantia nigra", "pituitary gland" and "retina". Protein groups with high expression in several brain tissues were also reduced to a single brain-level classification. The protein groups were then put into the three following categories: (i) Peripheral, (ii) Brain and (iii) Brain-only. Category i included proteins with high expression in all tissues except the brain, ii , proteins with high expression in the brain (but potentially in the peripheral tissues as well) and iii proteins with high expression exclusively in the brain (but potentially medium or low expression in the peripheral).
